# Can New Chemistry Make EVs That Charge in 5 min or Less?

**DOI:** 10.1021/acscentsci.5c00753

**Published:** 2025-05-06

**Authors:** Sam Lemonick

## Abstract

Battery start-ups and larger laboratories scrutinize materials in every part of electric vehicle systems, especially anodes.

## Graphical abstract

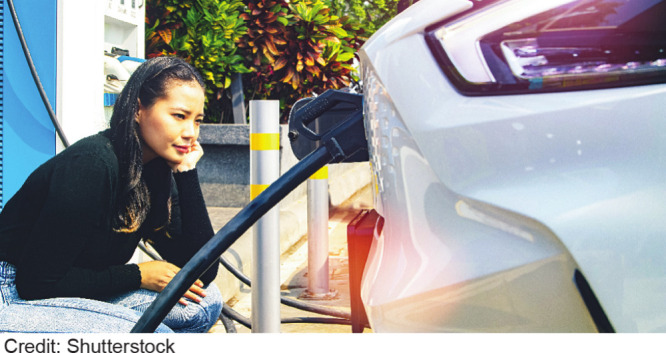

A time traveler visiting from the 1960s might be somewhat disappointed by the state of technological progress in 2025. There are no flying cars, no space hotels, no robot butlers.

But even though they cannot fly, cars are going through a major transformation from loud, jerky, smelly gas guzzlers to sleek electric vehicles (EVs) that quietly hum along roads. Nearly one in five new cars sold in 2023 were electric or plug-in hybrids, according to an International Energy Agency report.

The thing holding back EV adoption right now—besides the relatively high sticker price—is how long it takes to charge the battery, according to industry analyst Sam Adham of the research firm CRU Group.

EVs charge slowly. For some owners, the only practical way to charge is being parked for hours—say, overnight or while they’re at work. That is very different from the gas and diesel vehicles people are used to, which can be refueled in 5 min or so. The fastest chargers can add enough juice in 15 or 20 min to get someone to their next stop, but that is not the same as filling up the tank.

Credit: Shutterstock.

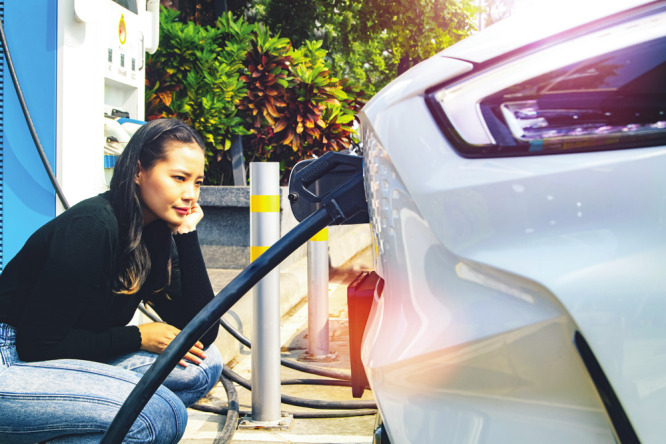

For someone living in an apartment building or a dense city neighborhood, without a place they can charge overnight, owning an EV might not be practical if they cannot charge it quickly. Likewise, a delivery company might not be able to justify a fleet of electric vans that have to charge for hours when they could be moving products.

It did not used to be this way. “Ten years ago, no one cared about fast charging,” says Venkat Srinivasan, a battery researcher at Argonne National Laboratory. The accepted definition of fast charging has changed in the past decade, he says: 15 min used to be considered fast enough, but car buyers are now demanding 5.

Fast battery charging is also a benefit to the companies that make charging stations. Faster charging means they can sell more energy to more customers in the course of a day. (Many companies also price fast charging higher.)

Making a battery that charges faster is not simple; it requires understanding the shortcomings of each component down to the molecular level. Nor is it easy to scale up a new battery’s manufacturing to millions of units. Experts agree it is unlikely that one battery chemistry or technology will emerge to fit every need.

“There’s no ‘perfect’ battery for electric vehicles yet, and honestly, there might never be one single ideal solution,” says Chong Yan, a battery researcher at the Beijing Institute of Technology. “It’s all about trade-offs,” which can include lifespan, safety, environmental impact, and vehicle range and charging time, he says.

In simple terms, a battery consists of two electrodes and a conductive material between them. When a battery is hooked up to a circuit—whether that is a flashlight bulb or an electric car motor—redox reactions inside the cell move electrons and ions out of one electrode, known as the cathode, and into the other, the anode. In the recharging process, these reactions are reversed.

**Figure:**
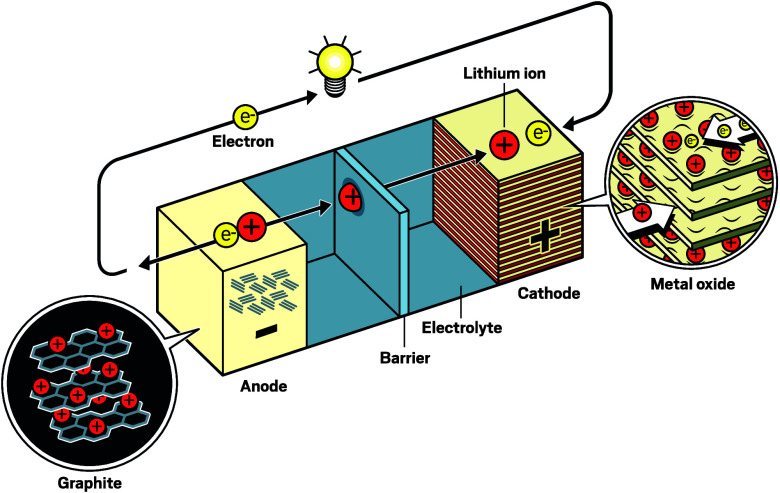
Lithium-ion batteries create electricity by moving electrons through a circuit, but inside the battery, lithium ions move from the cathode to the anode. The cathode is usually made of a layered metal oxide which fits ions between its layers. The anode has historically been made of graphite. Credit: Johan Jarnestad/The Royal Swedish Academy of Sciences/C&EN

The cathode has not seen radical changes over the years. Two of the most widely used classes of cathode materials—layered oxides, such as lithium nickel magnesium cobalt oxides (NMC); and polyanion oxides, like lithium iron phosphate oxides (LFP)—are descendants of the ones that John Goodenough, Arumugam Manthiram, and their colleagues developed at the University of Oxford and the University of Texas at Austin decades ago. These cathode materials work relatively quickly.

Getting the lithium ions to dissolve in the electrolyte and cramming them into the anode are significantly slower steps, Srinivasan says.

In addition to those components, rechargeable batteries in EVs require computers. The battery in an EV is not a single pair of electrodes but hundreds or thousands of cells wired together. Usually called a battery management system (BMS), the computer handles the flow of electricity to and from each cell to optimize performance and maintain the safety of the whole battery.

Most of the parts of the battery system are fair game for upgrades, and most of them have been improved since lithium-ion batteries came into production. At the moment, the ubiquitous graphite anode is probably getting the most scrutiny. “Graphite is the bottleneck,” says Ping Liu, a chemical engineer at the University of California San Diego.

Graphite’s layered structure and conductivity, as well as its low cost and toxicity, make it an obvious choice as an electrode material. But its 2D structure limits the paths that lithium ions can travel in and out. Argonne’s Srinivasan likens it to 1,000 people trying to leave a meeting hall through the same set of doors.

One solution could be to add more exits. Researchers at the US Department of Energy’s National Renewable Energy Laboratory have tried drilling holes in graphite as small as 5 μm wide with lasers. These channels give the anode more surface area for ions to enter and exit. In a patent, the researchers reported that they could charge a battery more than three times as fast when using the drilled-out electrode.

Others are looking for different materials. Silicon is a leading candidate. Like graphite, it is abundant, inexpensive, and poses little health or safety risk.

Batteries made with silicon can theoretically carry more energy in the same volume. Graphite can hold one lithium ion for every six carbon atoms, because the ions intercalate in the hexagonal pores in its sheets. In silicon anodes, the ratio is closer to 1:4.

One glaring problem with silicon is its lack of durability. A pure silicon anode swells in size by more than 300% when populated with lithium ions. A graphite anode, by comparison, grows by about 10% in volume.

Swelling can lead to cracking—not just of the anode but of the battery’s other components, and especially when the anode swells quickly, as happens in a fast-charging battery. That can damage the battery in the short term, but it can also reduce a battery’s overall lifespan, perhaps by years, Srinivasan says. Researchers do not yet have good real-world data to say with certainty how long batteries with silicon will last. And EV buyers might not want faster charging if it means replacing their battery pack—or their whole car—sooner.

It might be possible to get the best of both worlds using silicon-doped graphite. OneD Battery Sciences, founded in 2013, has developed a catalytic process that grows silicon nanowires inside the pores of graphite particles. The firm is now trying to license the technology to battery makers.

**Figure:**
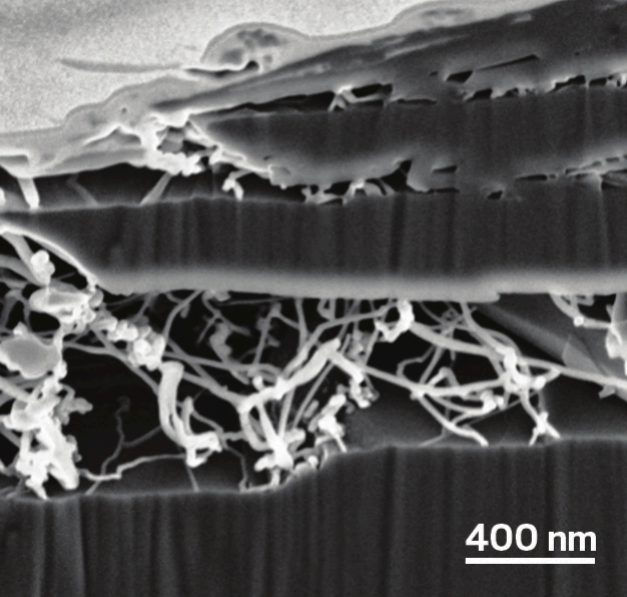
The company OneD grows silicon nanowires in between graphite’s layers to make anodes with the upsides of each material. Credit: OneD

CEO Vincent Pluvinage explains that in OneD's anodes, both the graphite and the silicon store lithium ions. The ions move about 4.5 times faster into and out of the silicon than they do with graphite, he says, which is what allows faster charging. At the same time, the strength of graphite ameliorates silicon’s swelling problems. Graphite also effectively moves current in and heat out. Heat can contribute to degrading the battery.

OneD opened a pilot plant to make its anode material in Washington state in 2024 and has announced a partnership with Koch Modular Process Systems to build a larger plant, though they have not said where or when. But given how long it takes for manufacturers to incorporate new technology into production vehicles, Pluvinage says, it will likely be at least 2030 before these batteries could make it into EVs.

Silicon is not the only option. UCSD’s Liu cofounded California-based Tyfast, which is working on a lithium vanadium oxide anode. The start-up received a grant from a US Department of Energy ARPA-E (Advanced Research Projects Agency–Energy) program called EVs4ALL, whose goal is a battery that can charge to 80% capacity in 5 min.

Metal oxide anodes, which several of the program’s grant recipients are exploring, have layers that can open up to let ions in and out more quickly, says EVs4ALL director Halle Cheeseman.

Tyfast’s lithium vanadium oxide batteries have lower energy density than the batteries currently used in EVs—meaning a pack with the same range would weigh more—but they charge very quickly. The company claims that its battery can fully charge in 10 min. Liu’s group attributes the speed to lithium ions rapidly hopping through the anode crystal between tetrahedral LiO_4_ sites and octahedral LiO_6_ sites.

**Figure:**
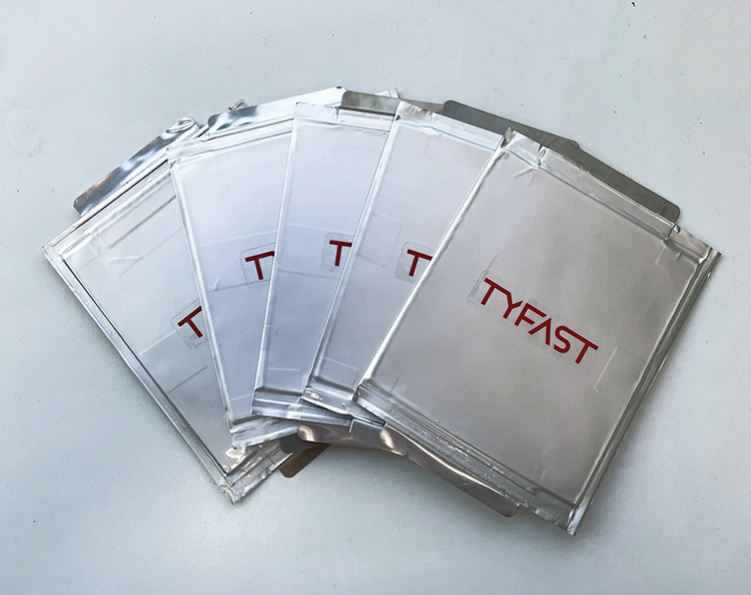
Tyfast's lithium vanadium oxide batteries charge faster than batteries with other anode materials but weigh more for the same amount of range. Credit: Ping Liu

While these bulkier batteries would reduce the range of passenger EVs significantly, Liu says their lower energy density would be less of a drawback in commercial vehicles. Trucks used in mining and other industries are already heavy, so a larger battery pack makes less of a difference.

Another group funded by EVs4ALL is exploring a glassy material. Project leader Anne Co of the Ohio State University would not share details but says the group, which includes collaborators at Honda and Argonne National Lab, has a paper in review. The team’s goal is to make smaller battery packs that have shorter range but charge quickly enough to be convenient.

Co says smaller packs would also help keep the vehicles affordable. But she acknowledges that battery makers outside the US have already beaten her group’s cost targets. It is a real question if batteries made in the US can be cost-competitive at all, she says.

EVs4ALL is also funding research on sodium- and potassium-ion batteries. Lithium has been the first choice for rechargeable batteries in large part because it is so light. Heavier elements mean heavier battery packs and less range.

Cheeseman says that while both sodium and potassium batteries charge quickly, sodium ones do not hold enough energy for today’s EVs. But potassium-ion batteries look more promising: they pack more energy than sodium-ion batteries and can charge more quickly than lithium-ion batteries because potassium ions diffuse faster through electrolyte.

Battery makers are also improving charging performance without necessarily introducing new materials. For instance, the Chinese battery company CATL announced last year that it was using an old chemical in its cathodes but processing it in a new way: pressing powdered LFP onto conductive foil rather than binding LFP particles together with adhesives. By making the electrode thinner, CATL reduces the electrical resistance, according to Adham, the industry analyst.

CATL has not disclosed details about the battery, which is called Shenxing Plus, but claims that it can recharge 600 km of range in 10 min at CATL’s proprietary fast-charging stations. That is 50% more range in the same amount of time than CATL’s last generation of LFP batteries, which the firm announced in 2023.

Other simple ways to speed up battery charging do not have that much to do with the battery materials themselves. Like most reactions at the lab bench, the reactions that allow lithium ions to move in and out of the electrodes happen faster at higher temperatures. Thus, the battery can charge faster when the battery is hotter—though unwanted side reactions that can degrade batteries also happen faster at higher temperatures.

Tesla enables this higher-temperature charging in a somewhat sneaky way. When a driver pulls up the navigation app to find the closest Tesla fast charger, the BMS computer lets the battery start to heat up more than it would under normal conditions. When the car pulls up to the charger, the warmer battery can be charged faster.

Another seemingly easy solution is to charge batteries at higher voltage, pumping more current into a battery. In March, the car and battery maker BYD announced a new charger that it says can give an EV a range of 400 km after being plugged in for 5 min. The 1,500 V system depends on silicon carbide (SiC) semiconductor chips in the car's on-board charging system. SiC chips can handle higher temperatures and voltages than chips made with silicon alone.

By way of comparison, Tesla’s fastest chargers operate at 1,000 V. BYD is one of many companies using SiC chips; its cars can also preheat their batteries before fast charging.

Using higher voltage does charge batteries faster, but it can also speed up battery degradation. Lithium ions flooded with electrons can convert to lithium metal that deposits on an electrode’s surface. (Although researchers understand the conditions under which this happens, they are still working on understanding exactly why.)

Those deposits are usually irreversible, meaning that less overall lithium and therefore less energy is available to move between the electrodes. And in the worst case, these deposits form spiky structures called dendrites that can pierce the barrier between the cathode and anode and short-circuit the battery.

BYD’s software likely mitigates the damage enough to ensure the battery’s long-term health, according to Adham. He says the new chargers may be meant primarily as a demonstration of the advantages of BYD’s LFP battery packs and not something most drivers will be using in the near term. Charging at 1,500 V would not be widely available until new infrastructure was built.

One last battery component that researchers are trying to optimize is the electrolyte solution that ions pass through as the battery charges and discharges. The Beijing Institute’s Yan focuses his energy on perfecting these ion solutions, because solvents can be a bottleneck. During fast charging, if lithium ions cannot move smoothly through a solvent, they create traffic jams and build up just outside the anode.

Lighter cosolvents, such as methyl acetate or ethyl acetate, can improve performance. A 2023 study from Dalhousie University found that using methyl acetate as the solvent for LiPF_6_—a common salt in electrolyte solutions—had doubled the conductivity of a conventional electrolyte for fast charging.

Argonne’s Srinivasan welcomes these potential improvements in chemistry—some commercially impractical, some incremental, and some more promising. He tries to be realistic about what the path to the 5 min fill-up will be.

Zooming out, he predicts that the solutions will come in three phases: First will be software, such as Tesla’s preheating. Second will be architecture, such as CATL’s pressed LFP powder. The third, which seems not to have arrived quite yet, will be new materials and new chemistry.

*Sam Lemonick is a freelance contributor to* Chemical & Engineering News, *the independent news outlet of the American Chemical Society.*

